# Clinical Lecture

**Published:** 1890-05

**Authors:** Henry M. Lyman

**Affiliations:** Chicago, Ill.; Professor of Physiology and Diseases of the Nervous System in Rush Medical College, Chicago


VOL. VII. MAY, 1890. No. 3.

### Original Communications.

### CLINICAL LECTURE.

By HENRY M. LYMAN, M. D., CHICAGO, ILL.
Professor of Physiology and Diseases of the Nervous System in Rush Medical College, Chicago.

### CEREBRASTHENIA—ASTHENIC MANIA—ALCOHOLIC INSANITY.

GENTLEMEN—The first patient that appears before you to-day is a shoemaker by trade, and is sixty-five years of age. He complains of indigestion. His daughter informs us that during the last few days he has been subject to dizziness, with a slight cough, and there is considerable twitching in the muscles of the face. This began about three days ago. His behaviour now is like that of a person with delirium tremens.

There is a marked distinction between his condition and that of a person suffering from meningeal inflammation, or we might suspect the occurrence of meningitis. But in meningitis there is fever; the pulse is somewhat accelerated, though not very considerably till the later stages of the disorder. It is usually about 100 and the temperature ranges from 101° to 102½°, with all the phenomena of fever present; but we are told that this patient did not have fever, and the statement that his behaviour is like that of a person with delirium tremens is a significant one. His condition is doubtless due to debility. The delirium is the result of disordered cerebral action, but it is not attended with high fever or with evidence of acute inflammatory disease. We have evidence of great depression. The nerves are weak and the nerve centers so much debilitated that they cannot transmit force to the muscles in sufficient quantity to keep the muscles still, and the consequence is that trembling condition which you see. The limbs tremble; the man is unsteady on his feet; in short, everything points to a condition of great prostration.

In the case of a patient who has acute meningitis, who is delirious perhaps, the muscles are in a high state of vigor. They do not shake; the limbs do not tremble if he undertakes a movement; he makes it with great firmness and energy. Such patients manifest a power of movement which is entirely different from the condition of the patient described here.

With regard to the disturbance which has occurred during the past few days, we cannot say anything; we have not knowledge enough. His daughter thinks it was connected somewhat with the medicine he took. That is possible. It is not improbable that he may have taken some narcotic drug or neurotic remedy that affected the brain. There are drugs, such as *hyoscyamus*, *belladonna*, *cannabis n dica*, etc., which, when administered to patients in large doses, will make them delirious and actually insane for the time being. If a patient were in an enfeebled condition, he would be more sensitive to the action of such remedies and would be more profoundly affected than if they were given in ordinary doses to a vigorous perso n. If the patient received a standard dose, such as is ordinarily administered, the physician is perfectly justified in giving medicine in that way, but he may not have had an opportunity of observing the condition of the patient or of learning his peculiar susceptibility to the effects of certain medicines, as that can only be found by experiment. As we know so little about the case, and do not know what drugs were administered, it is useless for us to speculate whether it was a certain drug or drugs that produced the disturbance. The thing that principally concerns us is the present condition of the patient.

It seems that for a month or more he has been suffering from debility, which commenced with dizziness and evidences of cerebral disturbance. His mind is somewhat affected and he is unable to take care of himself. What lesson may we learn from his case? In the first place, old age is a predisposing cause of debility and of cerebral disturbance. We not infrequently find that when old people become ill from any cause, a disturbance of the brain manifests itself, so that they are delirious. It is a common thing, when they are sick, to find them manifesting symptoms of great prostration. The pulse is weak, the heart beats feebly, and the muscles tremble. They talk at random; when they sleep they do so imperfectly; they dream a great deal; their condition is virtually that of a person with delirium tremens as a consequence of old age. Then, again, when a person who is under the influence of certain causes of disease suffers the disease, his disordered condition may give rise to a disturbance of the brain and derangement of the mind.

This patient has also been suffering from a catarrhal difficulty, and from a deficiency in sleep at night. His trouble began five or six weeks ago. Remember what influences were operative at that time. The influenza was in full force then in this city. A great many persons were suffering with just the class of symptoms of which he complains as a consequence of infection with the influenzal poison. The probability is that this man, predisposed by his age to nervous derangement, has suffered from influenza, and that as a consequence he manifests not only the phenomena of influenza, but those of cerebral exhaustion. He is now convalescing from the influenza, but as is true of all patients who have passed through it, a number of weeks are necessary for restoration to health, and I should not expect a patient who had had the influenza severely, especially one who is feeble, to recover his strength and vigor in less than six or eight weeks. I think you will find among your friends and acquaintances many persons who will tell you to-day that they have not been entirely well since an attack of influenza. They are attending to their business, it is true, but they do not feel as well as before the attack. The debility drags on. I think that is partly the condition of the patient before you, and that, together with his peculiar mental disturbance, is to be explained by the occurrence of old age and what might be called a toxæmia; for we find with such patients if they are subjected to a toxæmic condition or to the inhalation of ether or chloroform, or excess with alcohol, they will break down and suffer from symptoms of mental derangement. This sometimes happens in the case of women after childbirth, and we would then call their disorder *puerperal mania.* When it occurs from alcoholic excesses we call it *delirium tremens*, and after a surgical operation, *surgical insanity* or *surgical mania.* When it occurs after any one of the ordinary infectious diseases, and they are among the most common causes of it, such as scarlatina, diphtheria, pneumonia, influenza, then we would call it toxæmic insanity.

These cases almost always recover. Mental derangement originating in this way is very rarely a permanent thing. Cases of puerperal mania recover after three or four months. Cases of mania occurring after an injury, shock or surgical operation generally recover sooner or later. So is it with cases of this kind; they generally recover, though of course in elderly persons like *this man*, there is always reason to fear that they may relapse, or that the disease may tend to merge itself into *senile dementia.* You know it is not an uncommon thing with elderly people—and some persons are as old at sixty as others are at eighty—that when they get ill and feeble the atrophic condition of the brain becomes so marked that mental activity is impossible and the patient sinks into what is called senile dementia. That is the thing to fear in a case like this.

We had an interesting illustration of this form of cerebral disturbance some months ago in the hospital. There was an old man in the hospital who was suffering from abscess of the gall-bladder which had opened externally through the abdominal wall. There was a discharge from the abscess, and everything seemed to be going on without any trouble whatever. The patient was very comfortable and doing well. He was between 70 and 80 years of age. It so happened that one night there was brought into the hospital (by mistake perhaps) a maniac, who was placed in a room over the old man's bed. During the night he became violently excited, made a great deal of noise, tried to break the window, and the patients in the adjacent rooms were thrown into a state of considerable agitation. This old man began at once to manifest symptoms of mental derangement; he became delirious; he tried to get out of the window, and it became necessary to restrain him. He was speedily cured with opiates and stimulants.

Now the thing to do with these cases is to support them freely; give them plenty of good food to eat in quantities sufficient to steady their nerves, and the good that is to be obtained from stimulants will depend largely upon the previous habits of the patients. Give them full doses of opium. If the heart is feeble give them camphor with opium and capsicum. A grain of opium, a grain of camphor and a grain of capsicum make a good pill. Some physicians like to put in a little quinine. A half grain or a grain of quinine, with the 30th of a grain of strychnia, makes an excellent tonic. Under the influence of that tonic patients become quieted; their nerves become steady. It you feed them well they will sleep well. This is the line of treatment that is to be pursued. Sometimes they do not recover quickly. They remain in a state of insanity for many weeks or months, the attack coming on after an operation perhaps, and lasting two or three months before recovery takes place.

The London *Lancet* not long since described three or four of these cases occurring after surgical operations. Some of the cases recovered, but one patient was removed insane from the hospital. The patient was sent home really before the time for recovery. Very frequently it takes two or three months for restoration of sanity to take place, so you should not despair of recovery even if a patient does suffer for weeks and months after the commencement of the mental derangement. With careful nursing, supportive treatment, and anodyne remedies to relieve pain, and opiates are the best of all, patients will recover. That is the line of treatment I would recommend here.

We further learn that occasionally this old man goes on a spree. A person who suffers in this way is an inebriate. The common idea of drunkenness is excess in the use of liquor and it is associated with the continuous use of it, but there is a class of persons who perhaps have no fondness for the taste of alcoholic drinks. It is not a love for liquor that makes them drink, for the greater part of the time they do not drink at all. Sometimes they go two or three weeks, or six months, or longer, without drinking. Then comes on a paroxysm in which they commence to drink liquor if it is possible to obtain it. If they cannot get it at one place they will get it at another. If they cannot get one kind of liquor they will drink another. After several days the paroxysm will stop abruptly, perhaps, and they will not drink again for a long period of time, then the paroxysm comes on again ; yet, when you question many of those patients in regard to their drinking, they will tell you that they do not drink because they love liquor, but because they cannot resist the inclination. These paroxysmal drinkers constitute this class of inebriates, and the cause is doubtless attributable to a diseased condition of the nervous system.

There has been great misapprehension in regard to this matter among physicians. It is only of late years that it has been fully recognized as a nervous disease. We frequently hear of so-and-so who gets drunk and then stops drinking for a while. People forget that it is due to a nervous weakness, and they often wonder why he cannot restrain himself better than he does. His behavior is the result of his disease, just as the convulsions of an epileptic are the result of his disease. For instance, here we have an epileptic patient. He looks, talks and walks as steadily as anybody else, but now and then he has an epileptic paroxysm in which he lies in a state of insensibility for an hour, and when the paroxysm has subsided he goes to work and resumes his ordinary course of life for six weeks or two months, as the case may be, then comes another paroxysm. It is not a desire on his part for convulsions that brings on the paroxysms. They come because the patient cannot prevent them. Another thing: It shows the parallelism between this disease and epilepsy. Inebriates are sometimes sober, industrious, peaceful citizens in the intervals between the paroxysms. Sometimes when they drink they have a paroxysm of alcoholic insanity, which is one of the varieties of mania laid down in some of the text books as a form distinct from any other kind of insanity. There is nothing peculiar about it so far as the insane manifestation is concerned. It is connected with alcoholic excess, hence it is called alcoholic insanity. During this form of insanity patients will sometimes commit the vilest deeds—acts which they never would have done under any other circumstances, but which are the consequence of the paroxysmal mania under which they labor. The paroxysm may be of short duration, lasting a few hours perhaps, then it will pass away and they will entirely recover, just as a patient recovers from delirium tremens. In this condition they are known to commit heinous crimes. Assassinations, murders, are sometimes committed during these paroxysms. When a person is taken into court accused of murder during intoxication it is urged by the lawyer who defends him that he was drunk at the time the act was co mmitted, that that was the reason he committed murder. It is no excuse. The law does not exempt a man from the punishment of a crime because of drunkenness. He is punished for getting drunk. But in the attacks of insanity that occur after alcoholic excesses, such a person is as insane and as irresponsible as the person who is insane after an epileptic paroxysm. And the condition of patients with epileptic mania, or alcoholic mania differs in an important particular from the ordinary form of mania. After the paroxysm is all over he has but an imperfect recollection of what took place at the time; while the ordinary maniac is perfectly aware of what took place. He reasons about it, talks about it. There is not that loss of memory in connection with the event that there is in the case of epileptic mania or alcoholic mania.

This man's daughter tells us that she has suffered in years past from nervous prostration; that after nursing her baby she was greatly exhausted, and that exhaustion manifests itself in the way of difficult locomotion. She would fall easily. She had palpitation of the heart. While she may not have been as bad as represented, still she was on the way towards the establishment of complete insanity evidently—low spirited, and completely melancholic. Palpitation of the heart is an evidence of nervous weakness and irritability. You will find in the course of your practice certain women who are scarcely able to nurse their babes. They have only enough nerve force to carry them through an ordinary day's work, and when a little strain comes on, like nursing, bad news, or sickness in the family, then their hearts begin to palpitate, they become unsteady, low spirited, melancholic, insane. This woman has not got so far as that, but she has a tendency in that direction whenever extra pressure is brought to bear upon her.

As to the treatment of her condition, we should try to strengthen her as much as possible by giving a plenty of good blood-making food. She looks pale; her lips are pallid; the brain and spinal cord do not get that supply of red blood which they need for a full sufficiency of the organs. She should eat plenty of meat, and should have quinine, iron, and strychnine. The compound elixir of quinine, iron, and strychnine would be a good thing to be used for a year or more. The trouble with these patients is that they do not take treatment long enough. They take medicine for two or three weeks or a month and then disappear. We see no more of them, and if they do not get entirely well they are apt to attribute it to the medicine. A year would not be too long for such patients to take medicine. Along with restorative medication you must always look to the condition of the kidneys. Failures from a good diet and tonic regimen are often the consequence of neglect of the excretory organs. The bowels do not move often enough; the liver is not sufficiently energetic; the kidneys are deficient in their action. The bowels should move freely, if necessary with laxatives, like aloes, and the kidneys should be stimulated with small doses of bitartrate of potassium.

Such perversions of nutrition as you here observe are frequent.

It seems sometimes as if insanity involved not merely the intellectual functions, but the organs of nutrition and of circulation. The palpitation of the heart which patients experience you are sometimes tempted to liken to an insanity of the heart. The failure of digestion, or *nervous dyspepsia*, as it really should be called, is so obstinate, so persistently refractory under the influence of treatment, that you are reminded of the insanity of the brain with which you have been dealing. I am sometimes tempted to call it *insane dyspepsia*, a condition of things entirely different from the ordinary catarrhal dyspepsia that yields to treatment addressed to catarrhal conditions of the mucous membrane.

